# Chitosan-Stabilized Ag Nanoparticles with Superior Biocompatibility and Their Synergistic Antibacterial Effect in Mixtures with Essential Oils

**DOI:** 10.3390/nano8100826

**Published:** 2018-10-13

**Authors:** Ludmila Otilia Cinteza, Cristina Scomoroscenco, Sorina Nicoleta Voicu, Cristina Lavinia Nistor, Sabina Georgiana Nitu, Bogdan Trica, Maria-Luiza Jecu, Cristian Petcu

**Affiliations:** 1 Department of Physical Chemistry, University of Bucharest, Bucharest 030018, Romania; ocinteza@gw-chimie.math.unibuc.ro (L.O.C.); scomoroscencocristina@gmail.com (C.S.); 2 Department of Biochemistry and Molecular Biology, University of Bucharest, Bucharest 030018, Romania; sorina.voicu@bio.unibuc.ro; 3 National Institute for Research & Development in Chemistry and Petrochemistry ICECHIM Bucharest, 202 Spl. Independentei, Bucharest 060021, Romania; lc_nistor@yahoo.com (C.L.N.); sabina.nitu@yahoo.com (S.G.N.); trica.bogdan@gmail.com (B.T.); biotehnologie@icechim.ro (M.-L.J.)

**Keywords:** Ag nanoparticles, antibacterial, essential oil, synergistic effect

## Abstract

Silver nanoparticles (AgNPs) are considered a promising alternative to the use of antibiotics in fighting multidrug-resistant pathogens. However, their use in medical application is hindered by the public concern regarding the toxicity of metallic nanoparticles. In this study, rationally designed AgNP were produced, in order to balance the antibacterial activity and toxicity. A facile, environmentally friendly synthesis was used for the electrochemical fabrication of AgNPs. Chitosan was employed as the capping agent, both for the stabilization and to improve the biocompatibility. Size, morphology, composition, capping layer, and stability of the synthesized nanoparticles were characterized. The in vitro biocompatibility and antimicrobial activities of AgNPs against common Gram-negative and Gram-positive bacteria were evaluated. The results revealed that chitosan-stabilized AgNPs were nontoxic to normal fibroblasts, even at high concentrations, compared to bare nanoparticles, while significant antibacterial activity was recorded. The silver colloidal dispersion was further mixed with essential oils (EO) to increase the biological activity. Synergistic effects at some AgNP–EO ratios were observed, as demonstrated by the fractionary inhibitory concentration values. Our results reveal that the synergistic action of both polymer-stabilized AgNPs and essential oils could provide a significant efficiency against a large variety of microorganisms, with minimal side effects.

## 1. Introduction

Antibiotics have been very effective in controlling bacterial infections in the early part of the last century, thus being considered one of the most important findings in medical practice. Unfortunately, their inappropriate use has rapidly resulted in the appearance of antibiotic-resistant pathogens. Since the first infection with methicillin-resistant *Staphylococcus aureus* (MRSA) was reported in the United Kingdom in 1962, the phenomenon of resistance has worsened, because now there are already bacteria resistant to nearly all available antibiotics [1]. With increasing concerns of microbial infections, there is a growing interest in the development of new, effective antimicrobial agents. The former strategy of combatting resisting microorganisms was based on the development of new antibiotics, but this way has been abandoned by many pharmaceutical companies due to financing and regulatory difficulties, aggravated by the rapidity with which the resistance to newly synthesized antibiotics is developed [2,3].

Nanoparticles with antibacterial properties have been extensively studied during the last decade [4], with particular interest in the mechanisms of action against the multidrug-resistant strains of bacteria [5]. This has promoted research in the well-known activity of silver ions and silver-based compounds, including silver nanoparticles [6]. Silver is an excellent antimicrobial agent because of its broad-spectrum biocidal activity and limitation of the development of resistant microbial strains [7,8,9]. Its antiseptic properties have been known since antiquity. Currently, it is widely used in ionic-silver-releasing materials in medical devices, wound dressings, surgical equipment, personal care products, cosmetics, sporting goods, food and water storage, water filters, foods packaging, and so forth. Topical ointments containing some form of ionic silver are also used in many preservative applications (i.e., burn-associated injuries, eczema, etc.) [8,10]. Likewise, metallic silver is used in nanoparticulated materials, which naturally dissolve and oxidize in biological media, rendering partially oxidized nanoparticles with chemisorbed ionic silver on their surfaces.

Silver nanoparticles (AgNPs) can be synthesized by different physical and chemical approaches, but these methods are highly reactive and may pose a high threat to the environment [11,12]. Common methods involve the chemical reduction of silver salts by different reducing agents such as NaBH_4_, sodium citrate, or ascorbic acid [13,14]. However, in such chemical processes, the nanoparticles tend to aggregate due to the high surface energy inherent to the synthesis. In all of the methods, a noble metal precursor must be reduced to its metallic form first and then the formed metallic atom cluster must be able to form a stable sol. To inhibit the irreversible coagulation of particles, a stabilizing agent is usually required [15]. Both the stabilizer and the reducing agents used for the preparation might exhibit environmental toxicity [16].

Preparation of AgNPs by green synthesis approaches has advantages over conventional methods involving chemical agents associated with environmental toxicity. Green synthesis methods include mixed-valence polyoxometalates, polysaccharides, Tollens process, irradiation, and biological components. The mixed-valence polyoxometalates method involves the use of polyoxometalate derivatives which act as both a reducing agent for silver ions and a capping agent for the nanoparticles produced [17]. The Tollens process involves the reduction of Ag(NH_3_)^2+^ by saccharides forming AgNP films with particle sizes from 50–200 nm, Ag hydrosols with particles in the order of 20–50 nm, and Ag colloid particles of different shapes [18]. AgNP synthesis by irradiation of Ag^+^ ions does not involve a reducing agent and is an appealing procedure [19]. Ecofriendly plant extracts contain proteins and other active components, which act as both reducing and capping agents, and the formation of stable and shape-controlled AgNPs was also reported [20]. Electrochemical synthesis for the fabrication of AgNPs is one of the most used methods, especially for industrial purposes, since it is an ecofriendly, cost-saving, and facile technique to generate localized nanostructures. The possibility of selecting the experimental parameters allows a high degree of control of size and characteristics of the generated nanostructures [21].

The antibacterial activity of Ag nanoparticles, similar to other metallic nanomaterials, is closely related to size and surface functionalization [22].

Exposure to nanoparticles for medical purposes involves intentional contact or administration; therefore, understanding the properties of nanoparticles and their effect on the body is crucial before clinical use can occur [23]. The most important limitation in the widespread application of Ag nanoparticles in therapy is the public concern regarding their toxicity [24]. Physical parameters such as surface area, particle size, surface charge, and zeta potential are very important for providing mechanistic details in the uptake, persistence, and biological toxicity of nanoparticles inside living cells [25,26]. Recently, many studies have indicated that environmental factors, such as ionic strength, pH, or ionic type could also induce transformations of AgNPs that are expected to affect their properties [27,28]. These findings suggest that the toxicology research of nanomaterials should also consider conditions when AgNPs coexist with and without their bioavailable ions [29].

As a result, various studies have been designed to assess the effect of even low doses of silver nanoparticles (AgNPs) on bacterial cell membranes [30,31,32]. Results on in vivo toxicity of AgNPs have been reported for various living organisms, such as zebrafish [33,34], catfish [35], common carp [36], *Corbicula fluminea* clam [37], *Caenorhabditis elegans* worm [38], and mice [39,40]. The interaction of metal nanoparticles with nucleic acids is also a hot topic in the field of bioinorganic research, due to the interest in elucidating possible effects of Ag nanoparticles on the synthesis, replication, and structural integrity of DNA and RNA [41] and the mechanism of DNA–nanoparticle binding [42]. Several bio-organic compounds have been used for Ag nanoparticle surface functionalization, in order to reduce the toxicity by mitigating the interaction with normal living cells [43,44].

Synergistic or antagonistic effects of Ag nanoparticles with different antibacterial compounds used in the treatment of infections were reported more than 20 years ago against a wide range of bacteria, including certain antibiotic-resistant bacteria [45]. Unfortunately, the lack of suitable experimental design results in many cases in a qualitative, not quantitative approach, and the reported synergistic effect is questionable [46].

In the last decade, essential oils originating from various plant species have also been investigated for antibacterial properties, as a possible solution to reduce antibiotic use [47]. The combination of essential oils with antibiotics or other antibacterial compounds was found to be effective against most common bacteria and fungi, but studies on the essential oils–Ag nanoparticles synergism are limited [48].

In the present study, we considered the electrochemical method as a simple, fast, and inexpensive technique to produce colloidal silver with suitable size and size distribution. The rational design of novel AgNPs stabilized with biopolymeric compounds was utilized to ensure a stable, nontoxic metallic Ag nanostructure that combined with essential oils could generate an improved antibacterial efficiency.

## 2. Materials and Methods 

### 2.1. Reagents

Silver bars used as electrodes were commercial products (99.999% purity) with standard dimensions of 80-mm height and 2-mm diameter, kindly donated by SC Sonnenkreuz SRL, Brasov, Romania. Chitosan of high molecular weight (mol wt 310,000–375,000 Da) and chitosan of low molecular weight (mol wt 50,000–190,000 Da) were purchased from Sigma-Aldrich (Louis, MO, USA).

Essential oils from thyme (*Thymus vulgaris*) and clove (*Szygium aromaticum*) were purchased from Young Living Essential Oils (Lehi, MT, USA) and were used without further purification. The oils were stored at 5 °C, and stock solutions in dimethyl sulfoxide (DMSO) were prepared and used in the same day to minimize volatilization and change in chemical properties.

The other reagents used in the study were analytical grade, commercially available, and used without further purification. For particle synthesis, distilled water produced with a Merit water still W 4000 (Bibby Scientific LTD, Staffordshire UK) was used. The distilled water had pH 4.7 and conductivity 2.3 µS.

### 2.2. Methods and Instrumentation

Synthesis of Ag nanoparticles: AgNPs were synthesized using an electrochemical method with a sacrificial anode [49]. The synthesis was adapted for commercially available instruments in order to design an easy scalable and cost-saving method able to be implemented in industry. The used device was a laboratory Colloid Master 1000 model, (Colloidmaster GmbH, Isselburg, Germany), based on “True ppm” technology patented by the company. The instrument operates with automatic settings of time and current intensity applied, in order to produce standardized Ag dispersions at nominal concentrations of 6, 10, 25, 50, and 100 ppm. In the present study, a preliminary experiment was performed on a sample prepared in conditions to obtain a nominal concentration of 6 ppm, in order to confirm the Ag concentration using the inductively coupled plasma - optical emission spectrometry (ICP–OES), using the instrument Optima 2100 DV ICP-OES (Perkin Elmer, Waltham, MA, USA). A value of 5.83 ppm was found.

The two silver bars working as electrodes were connected to the voltage source of the instrument and placed in a 250-mL glass beaker. An adequate volume of dispersing fluid (distilled water or stabilizer solution) was added. In the modified synthesis method, solutions of various concentrations of stabilizing agents were used to replace the water. The reaction was performed for a period of time ranging from 1 h to 4 h, at room temperature, in the dark, and without stirring. Samples with various concentrations (10, 25, 50, and 100 ppm) were prepared. Further characterization of products revealed no significant variation in the shape and size of the particles; thus, 25 ppm samples were used for further studies.

### 2.3. Characterization of Nanoparticles

The optical properties of the silver dispersions were characterized by using ultraviolet-visible (UV-vis) spectroscopy. The measurements were performed on a Jasco W530 spectrophotometer in quartz cuvettes, in the range 200–900 nm.

Particle size distribution and the average hydrodynamic diameter were measured by dynamic light scattering (DLS) techniques, using a ZetasizerNanoZS ZS ZEN 3600 instrument (Malvern Instruments Ltd., Malvern, UK) and disposable square polystyrene cuvettes DTS0012 (Malvern Instruments, Malvern, UK). The determinations were performed on the AgNPs suspensions (as obtained from synthesis). The same instrument, the Zetasizer Nano ZS ZEN 3600, was used to perform zeta potential measurements, based on the electrophoretic mobility measurement technique of laser doppler velocimetry (LDV). Folded capillary cells DTS 1060 (Malvern Instruments, Malvern, UK) were used for zeta potential analysis.

Samples were also analyzed by transmission Fourier transformed infrared spectroscopy (FTIR), using a Tensor 37 (Bruker, Munich, Germany) and the potassium bromide (KBr) pellet technique. The measurements were recorded in the 4000–400 cm^−1^ range. The KBr pellets were produced as follows: a volume of approximately 100 µL of AgNPs aqueous dispersion was added to ~2 g KBr; the mixture was dried in a vacuum oven at 70 °C for 6 h; the dry KBr powder, containing AgNPs, was then used to obtain pellets.

The morphology of the Ag nanoparticles was characterized using transmission electron microscopy. Bright field micrographs of the sample were obtained with a FEI TEM Tecnai G2F20 TWIN instrument (FEI, Eindhoven, The Netherlands) operating at an accelerating voltage of 200 kV. A few drops of the aqueous suspensions of the AgNPs (as obtained from synthesis) were dropped on Tedpella carbon-coated Cu grids. The excess liquid was removed by filter paper.

### 2.4. Quantitative Assay of Antimicrobial Activity

The antimicrobial properties of the AgNPs were tested against 3 reference microbial and 1 fungal strains: Gram-positive *Staphylococcus aureus* (*S. aureus* ATCC 6538) and Gram-negative *Escherichia coli* (*E. coli* ATCC 8739), *Pseudomonas aeruginosa* (*P. aeruginosa* ATCC 9027), and *Candida albicans* (*C. albicans* 10231).

The minimal inhibitory concentrations (MIC, ppm) were determined by binary serial microdilution assay performed in 96-well microtiter plates.

For media, buffer preparation, and dilutions, physiological sterile water was used. Incubation for antibacterial assays was done in a shaker–incubator at 37 °C. To the final volume, that included the liquid culture medium and chemical compounds, 10 µL of microbial suspension with a density of 10^6^ CFU/mL obtained from a suspension of 0.5 MacFarland (10^8^ CFU/mL) was added. Microbial suspensions were obtained using physiological sterile water and 24-h cultures obtained on simple agar.

After incubating the plates at 37 °C for 24 h, the results were obtained by reading optical densities at 620 nm using a spectrophotometer. A positive control without treatment (nanoparticles or essential oils) and negative control with liquid culture medium only were included in each experiment.

The MIC value of the silver dispersions is considered to be the concentration corresponding to the last well in which microbial growth was not observed.

In order to evaluate the synergism between the AgNPs and essential oils, quantitative parameters of antimicrobial activity were determined by the MIC method on selected bacterial strains, using AgNP–essential oil mixtures at various volumetric ratios. The other details of the method are preserved as previously described for Ag dispersion tests. The synergistic effect is analyzed by using the determination of the fractional inhibitory concentration index (FICI), calculated as the sum of the fractional inhibitory concentration for the two components of the mixture, FIC (A) and FIC (B), respectively [50,51,52]. The values of FICI are interpreted as follows: ≤0.5: “synergistic”, >0.5 and ≤1: “additive”, >1 and ≤4: “indifferent” and ≥4: “antagonistic” [50,51,52].

The concentration in the MIC experiments for the determination of the fractionary inhibitory concentration index was selected starting with the MIC values of individual components, and then the domain was refined, according to the preliminary FICI results. In the present study, the concentration for Ag colloidal dispersions was in the range 0.1–25 ppm, and for essential oils, was from 1–0.002%.

### 2.5. In Vitro Biocompatibility

The normal dermal fibroblast cell line CCD-1070 Sk (CRL-2091) obtained from the American Type Culture Collection (ATCC) was used to test the influence on normal cells of the exposure to the prepared AgNPs. The fibroblast cells were cultured in minimum essential medium (MEM) (Thermo Fisher, Gibco, Waltham, MA, USA) with pH 7.4, containing 2 mM L-glutamine, 1 mM sodium pyruvate, 1.5 g sodium bicarbonate, and 1% PSA antibiotic (penicillin, streptomycin, amphotericin), supplemented with 10% fetal bovine serum (Gibco, USA). The cells were developed in flasks of 75 cm^2^ and kept in a humidified atmosphere (95% humidity) containing 5% CO_2_ at 37 °C. For evaluation of the effect of silver nanoparticles on cell viability, dermal fibroblasts were seeded at a density of 5 × 10^4^ cells/mL in 24-well plates and incubated with different concentrations of nanoparticles for 24 h. Assessment of cell viability was performed using the 3-(4,5-dimethylthiazol-2-yl)-2,5-diphenyltetrazolium bromide (MTT) reduction assay. After the exposure interval, the cell medium was replaced with phosphate-buffered saline (PBS) containing 1 mg/mL MTT and incubated at 37 °C for 2 h. The absorbance was read at 595 nm using a microplate reader (Tecan, GENious, Grödic, Germany). Survival rate was expressed as a percentage of the control (untreated cells were represented as 100% viability). Experiments were performed in triplicate.

The cytotoxic effects of the tested AgNP samples on cells were evaluated using the lactate dehydrogenase (LDH) release assay. Measurements were performed by using a cytotoxicity detection kit (TOX-7, Sigma-Aldrich) according to the manufacturer’s instructions. The absorbance was read at 490 nm using a microplate reader Tecan Genios, with the optical density (OD) being directly correlated with the cell membrane lysis and cell death.

All results were expressed as mean ± SD (standard deviation) of three independent experiments. Statistical analysis was performed with GraphPad Prism software (Version 5; GraphPad Software, La Jolla, CA, USA) by one-way ANOVA test. The *p* values of less than 0.05 were considered statistically significant.

## 3. Results and Discussion

### 3.1. Synthesis and Characterization of Chitosan-Stabilized AgNPs

Silver nanoparticles were obtained using a facile, ecofriendly procedure based on the electrochemical method in order to avoid the use of harmful reduction reagents. The bare AgNPs were obtained in distilled water as the dispersion media, without any stabilization agent, and were further used as the reference material in the evaluation of the effect of surface functionalization on the interaction with cells.

**Figure 1:**
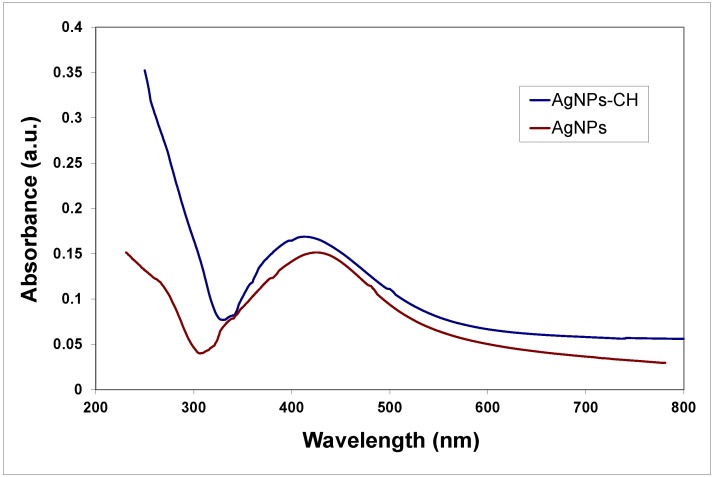
The ultraviolet-visible (UV-vis) spectra of silver nanoparticles (AgNPs) with and without chitosan (CH) stabilizer.

Polymers (various types of chitosan) were used for surface functionalization to prevent aggregation and increase the stability of the nanoparticles. The formation of the AgNPs in the reaction medium during the electrochemical synthesis was evidenced by measuring optical properties. Absorption spectra of bare and chitosan-stabilized AgNPs are presented in Figure 1.

In the spectra recorded for samples of AgNPs prepared in distilled water, both in the presence and absence of polyelectrolytes, absorption peaks at approximately 410 nm are present, corresponding to the characteristic surface plasmon resonance (SPR) signal of the AgNPs. The SPR absorption band is quite symmetrical and enlarged; the broadening of the signal could be the result of the formation of quasi-spherical particles or a rather large distribution of sizes. Further characterization of the shape and size of the nanoparticles was performed through TEM and DLS methods.

**Figure 2:**
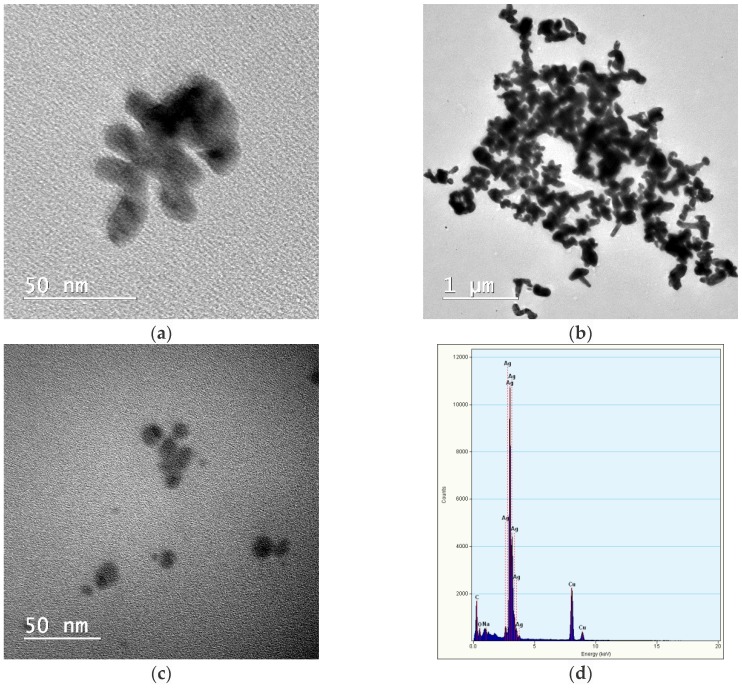
TEM images of AgNPs prepared in distilled water without stabilizer (**a**), with 0.05% chitosan as stabilizer (**b**) and (**c**), and energy dispersive spectroscopy (EDAX) for the AgNP sample with chitosan (**d**).

The morphology of the AgNPs was evaluated from the TEM images. Micrographs for the bare AgNPs prepared in water without stabilization compared to those stabilized with chitosan are shown in Figure 2.

In all samples, single metallic nanoparticles were present, together with clusters formed by smaller particles aggregates, embedded in the same polymeric shell when chitosan was used during the preparation.

As is expected from the electrochemical synthesis, the particles were quasi-spherical in shape and exhibited a high tendency to aggregate in the absence of stabilizing agents.

The AgNPs prepared using electrochemical methods are known to be more polydisperse compared to those prepared using chemical reduction methods, in which the control of size and polydispersity is more easily achieved through variation of reaction parameters.

**Figure 3:**
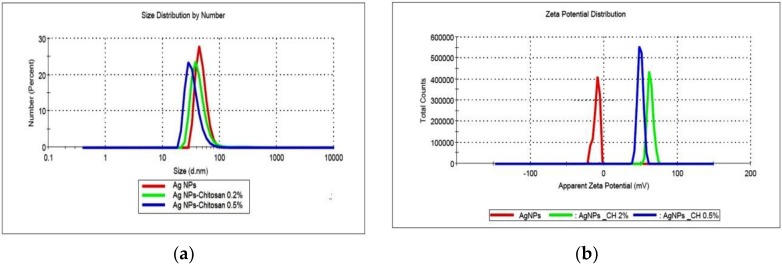
(**a**) The size and size distribution of AgNPs stabilized with high molar weight (HMW) chitosan solution; (**b**) Zeta potential of the same samples.

The size and size distribution of the products obtained in various conditions were determined using dynamic light scattering, and the results are shown in Figure 3.

Various samples of AgNPs prepared in different stages of this work were compared in terms of size and size distribution, considered as critical parameters for antibacterial activity. For example, six colloidal dispersions (50 ppm concentration) prepared in different periods exhibit an average size of 71.2 ± 8.8 nm.

In most of the cases, for the AgNPs synthesized without a stabilizer, a bimodal distribution of size is recorded; a small population of large aggregates of hundreds of nanometers in size could be observed in intensity mode. The stabilization with the polyelectrolyte chitosan (high molecular weight, HMW, or low molecular weight, LMW) was used to prevent the aggregation of particles. No major difference was noticed between the two types of polymers in the stabilization efficiency; changes in size and size distribution of samples were recorded during a period of 4 weeks of storage (data not shown). The AgNPs prepared in polymeric solutions exhibit smaller average size; for example, in the case of dispersions prepared at 25 ppm concentration, particle diameters were 61.9 nm and 65.4 nm for samples obtained in 0.5% and 0.2% chitosan solutions, respectively, compared to the value of 98.2 nm for the sample synthesized in pure water. The polydispersity index (PDI) ranges from 0.205 to 0.398, which is higher than the accepted value of 0.200, but is close to it nevertheless. Considering the specificity of the electrochemical method used in synthesis and the use of chitosan with medium molecular weight on average, the PDI obtained indicates good monodispersity for polymer-stabilized nanoparticles.

The zeta potential of bare AgNPs was negative (average value −11.2 mV). AgNPs stabilized with chitosan exhibited positive zeta potentials, with values larger than 50 mV and up to 75 mV, according to the degree of functionalization. The electrostatic repulsion between particles increases at higher values of zeta potential, and thus results in the improvement of the colloidal stability. The positive value of zeta potential is considered as an advantage in the interaction of the particles with the negatively charged bacterial wall, leading to a better internalization.

In order to evaluate the influence of the essential oils on the stability of chitosan-coated AgNPs, a short-term stability test was performed. Samples of chitosan-stabilized AgNPs with and without essential oils (20:1 ratio) were kept at room temperature in the dark, and size and size distribution was measured after 3 days using the DLS method (data not shown). No significant changes were recorded for the average size of particles, and the DLS diagram did not present additional signals showing larger aggregates.

**Figure 4:**
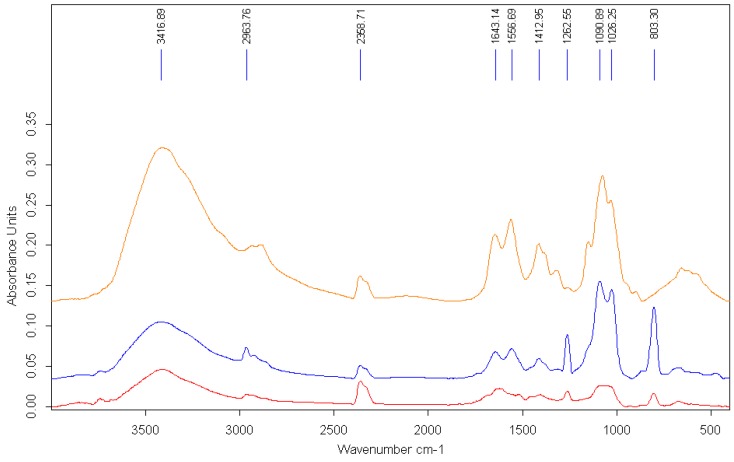
The FTIR spectra of AgNPs stabilized with chitosan solution (HMW) (yellow = pure chitosan; blue = AgNPs stabilized in 2% chitosan solution; red = AgNPs stabilized in 0.2% chitosan solution).

The interaction of AgNPs with the polymer is also evidenced by the modification of the FTIR spectra (Figure 4).

Representative peaks of the polymeric chain could be recognized in the FTIR spectrum of AgNPs prepared in chitosan solution that confirmed the functionalization of the surface of the metallic particles. The signal at approximately 3400 cm^−1^ in the pure chitosan spectrum, which corresponds to the –OH stretching, is broadened and slightly shifted due to the interaction with the AgNPs. Other specific peaks for chitosan are modified, either by reduction of intensity or shifting. The peak at 1652 cm^−1^ in the pure polymer spectrum, assigned to the –CONH_2_ groups, is significantly reduced in the spectra of AgNP–chitosan particles. The major changes in the bands at 1557 cm^−1^, corresponding to the amino groups of chitosan, shifted to a lower wavelength, suggesting that the mechanism involved in the stabilization of AgNPs with chitosan is based on the interaction of primary –NH_2_ groups with the nanoparticles’ surface [53].

### 3.2. Biocompatibility Assay

Since the major concern in using metallic nanoparticles for the fabrication of medical and personal care products is the reported toxicity, our approach is to improve the biocompatibility of AgNPs by coating with a polymeric shell. Thus, chitosan was selected to act both as a stabilizer and to decrease the toxicity of the particles. Chitosan is currently recognized as a highly biocompatible polymer, and is approved by the Food and Drug Administration (FDA) for wound dressing applications and for dietary use in some European countries and Japan [54]. Many papers have reported that the viability of normal cells after exposure to AgNPs decreases in a time- and dose-dependent manner, but no general conclusion could be applied as major differences have been recorded depending on the type of cells, protocol of the experiment, and size and functionalization of the Ag nanoparticles used. The most common use of Ag colloidal dispersions as antibacterial agents is as active compounds in creams, ointments, wound dressings, and hydrogels for topical administration. The normal cells with which the Ag nanoparticles interact in this case are dermal, thus the influence of the AgNPs on the viability of the normal dermal fibroblast cell line CCD-1070 Sk was evaluated as relevant for toxicity assessment.

Two types of chitosan were used, with high molecular weight (mol wt of 310,000–375,000 Da) and low molecular weight (mol wt of 50,000–190,000 Da), in order to study the influence of the polymer characteristics on the increase in biocompatibility of AgNPs functionalized with a polyelectrolyte.

**Figure 5:**
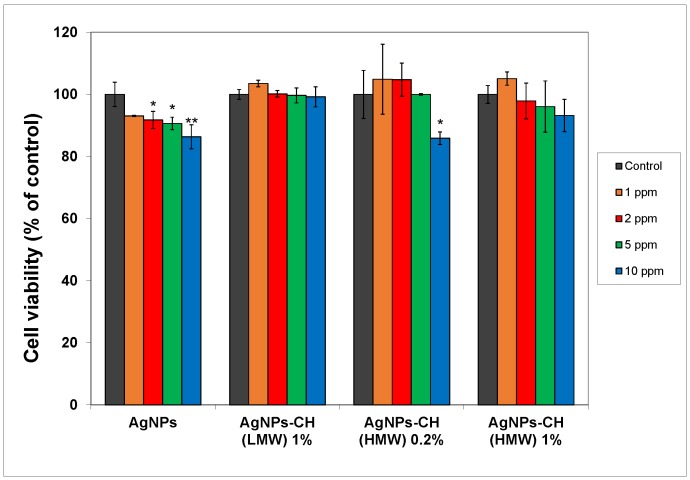
The viability of the CCD-1070 Sk cells treated with different concentrations of AgNPs after 24 h. The values are the means of the results for each sample from three separate experiments. Error bars indicate standard errors of the means. The statistical significance is indicated as follows: * *p* < 0.05.

In Figure 5, the MTT results are shown for the fibroblasts exposed to different concentrations of AgNPs (from 1 ppm to 10 ppm) stabilized with chitosan, compared to bare particles.

As expected, unmodified AgNPs produced reduction of the cell viability at concentrations higher than 2 ppm, of up to 86% compared to the control for the 10 ppm concentration. For the AgNPs stabilized with HMW chitosan, when using the 0.2% concentration of the polyelectrolyte, an increase in cellular viability was recorded for the concentration of particles up to 5 ppm. With the increase of the chitosan in the stabilizing solution, better functionalization of AgNPs was expected, leading to superior biocompatibility, as was observed in the results of the MTT assay. No significant changes were recorded in the viability of treated cells compared to the control across the whole range of AgNP concentrations used for HMW chitosan at 1% in the stabilizing solution. Similar results were obtained for the other type of chitosan used (LMW), with lower molecular weight.

The increase of the biocompatibility is due to both the reduction of the ionic Ag^+^ release and to the higher biocompatibility of the chitosan itself. The Ag^+^ could be trapped into the polymeric corona through Coulombic interaction with functional groups of the polyelectrolyte macromolecule (especially amino groups) [55].

**Figure 6:**
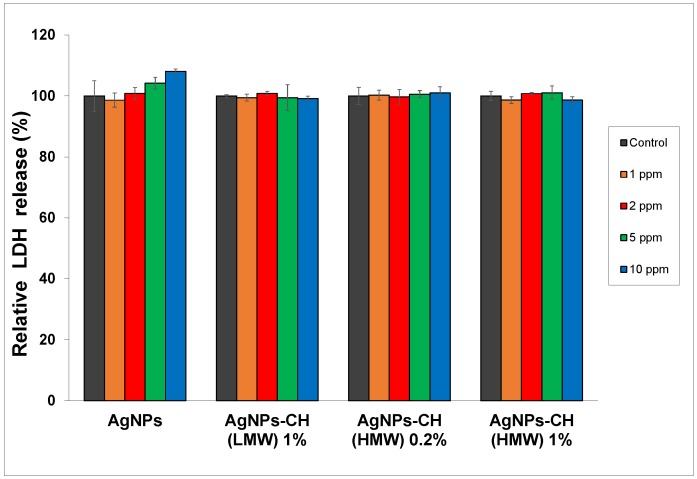
The lactate dehydrogenase (LDH) released in normal fibroblast cells, CCD-1070 Sk, treated with different concentrations of AgNPs after 24 h. The values are the means of the results for each sample from three separate experiments. Error bars indicate standard errors of the means.

In Figure 6, it can be seen that after exposure of the dermal cells to AgNPs, LDH activity did not reveal any significant change in cytotoxicity between treated cells versus control cells at concentrations ranging from 1 to 5 ppm.

For the cells exposed to bare AgNPs at the 10 ppm concentration, the integrity of the cell membrane was slightly affected, with LDH activity showing the highest increase of 8% compared to the control.

Taking these data into account, AgNPs stabilized with 1% chitosan could be considered safe for use in further experiments at concentrations up to 10 ppm.

### 3.3. Quantitative Assay of the Antimicrobial Activity and Synergistic Effects

Antibacterial activity of various formulations based on AgNPs was evaluated by using the microdilution method, measuring the OD of the samples via a microplate reader.

The results for the antimicrobial activity of the new AgNPs stabilized with 1% HMW chitosan, with average size of 62.5 nm, expressed in terms of minimum inhibitory concentration (MIC) against various microorganisms (bacteria and fungi) are presented in Table 1.

These results show that the tested AgNPs exhibited a specific antimicrobial activity, depending on the nature of the microorganism, which ranged between 0.78 ppm and 3.13 ppm.

The minimum inhibitory concentration (MIC) values are consistent with good antimicrobial activity of the AgNPs against all tested pathogens, taking into account the size of the samples.

No significant variation was recorded when chitosan-stabilized AgNPs were used compared to the unmodified Ag particles with similar sizes. The chitosan polymer is reported to possess an intrinsic antibacterial activity, but the results have been obtained mainly for chitosan nanoparticles or hydrogels. In our experiments on the inhibition of *E. coli* growth, both types of chitosan used in this study (high molecular weight, HMW, and low molecular weight, LMW) exhibited the same MIC value of 3.125%, which is far larger than the concentration of the polyelectrolyte present as the stabilizer in the AgNP dispersion.

**Figure 7:**
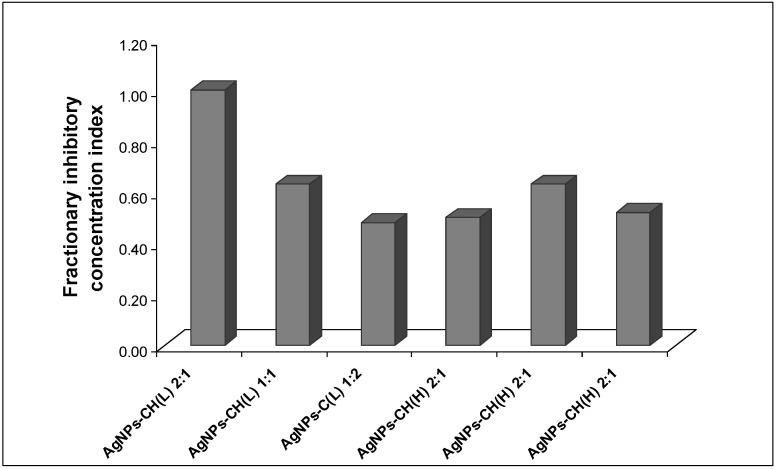
The variation of the fractional inhibitory concentration index of AgNP–chitosan solution mixtures measured for the inhibition of *E. coli* growth; CH(L) – low molecular weight chitosan; CH(H)—high molecular weight chitosan.

Nevertheless, a further study to investigate a possible beneficial effect in the case of using higher concentrations of chitosan mixed in the Ag colloidal dispersion was performed. The interaction between the Ag dispersion and the biopolymer chitosan was analyzed by computation of the fractional inhibitory concentrations index (FICI) from the MIC values of AgNP–chitosan mixture, according to the equation described in the literature [50,51,52] (presented in Figure 7). The interpretation of the results was performed according to the accepted criteria described in the previous section.

No synergistic effect was detected in the antibacterial effect against *E. coli* for both HMW and LMW chitosan, for all tested ratios.

The antibacterial assay for the mixtures of AgNPs and chitosan solutions at various ratios leads to values of the fractionary inhibitory concentration index (FICI) ranging from 0.50 to 1.00, which indicate an additive effect.

Another possible means of increasing the antibacterial efficiency of the AgNPs was investigated, by adding essentials oils (EOs) to the colloidal dispersion. The commercial essential oils are water-distilled extracts of various portions of the source plants *Szygium aromaticum*, in the case of clove oil, and *Thymus vulgaris* for thyme oil. Reported in the literature as being major components of the thyme and clove EOs used in the present study are eugenol (85.5%) and eugenol acetate (12%) in clove EO, and thymol (47.3%), p-cymene (18%), and carvacrol (11.8%) in thyme EO, respectively [56].

Essential oils exhibited inhibitory effects against various pathogens, usually at concentrations ranging from 0.02 to 0.3%, significantly lower than the concentrations that produce side effects, such as irritation and dermal allergies [57]. The minimum inhibitory concentrations (MICs) of the thyme and clove oils used were determined against the bacterial and fungal strains and are summarized in Table 2.

**Figure 8:**
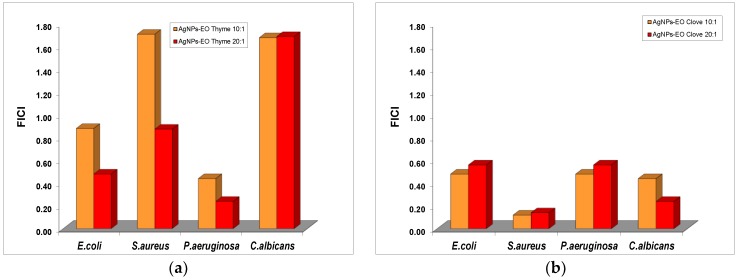
The variation of the fractional inhibitory concentration index (FICI) of bare AgNPs–essential oils (Eos) at various ratios. (**a**) Thyme oil; (**b**) Clove oil.

The impact of the addition of EOs from clove and thyme to the Ag colloidal dispersions without surface functionalization was evaluated, and the results suggest a significant decrease of the MIC values of silver nanoparticles in the mixture with both volatile oils (Figure 8).

For *C. albicans*, the formulations containing AgNPs and thyme oil exhibited only indifferent effect for both ratios tested, similar to the effect obtained for the ratio 10:1 against *S. aureus*. An additive effect was recorded in the antibacterial effect of AgNP and thyme oil mixtures against *S. aureus* and *E. coli*. Only for *P. aeruginosa* was a synergistic effect present at both 10:1 and 20:1 ratios.

The formulations with bare AgNPs and clove oil exhibited an additive effect in their antibacterial properties against *E. coli* and *P. aeruginosa*, while a synergistic effect was shown against *S. aureus* and *C. albicans*.

**Figure 9:**
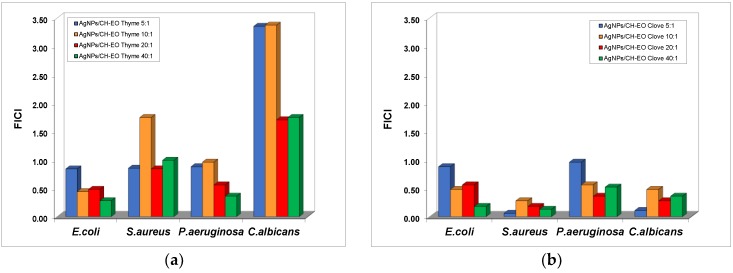
The variation of the fractional inhibitory concentration index (FICI) of mixed systems with AgNPs stabilized with 1% LMW chitosan and essential oils. (**a**) Thyme oil; (**b**) Clove oil.

The influence of the stabilization of AgNPs on the synergistic effect obtained in the presence of the essential oils was investigated and the results are summarized in Figure 9.

The addition of thyme oil to the dispersions of AgNPs stabilized with chitosan (the low molecular weight type) produces a decrease in the MIC values of AgNPs recorded for *C. albicans* and *S. aureus* strains, but the effects are indifferent for all the ratios ranging from 5:1 to 40:1. Synergistic effects were observed in the cases of *E. coli* and *P. aeruginosa*, but at mixtures less concentrated in essential oil compared to the formulation with bare AgNPs.

The mixtures with clove oils exhibited additive effects of antibacterial activity against *E. coli* and *P. aeruginosa*, with the sole exception of the AgNP–EO mixture at the 40:1 ratio for the *E. coli* bacterial culture. Exceptional synergistic effects were obtained for the inhibition of *S. aureus* growth with silver dispersions stabilized with chitosan in the presence of clove oil for all ratios tested.

Since the essential oils are very complex in composition and the interaction of various natural compounds with the polymeric coating of the metallic particles could also affect the biological properties, further studies are needed in order to elucidate the role of the chitosan stabilization in the apparent synergistic or additive effects.

## 4. Conclusions

In the present work, silver nanoparticles stabilized with various types of chitosan were fabricated by a simple, cost-efficient, environmentally friendly electrochemical method. The obtained nanoparticles were quasi-spherical in shape, with an average size of 65 nm and good monodispersity, as observed in a DLS diagram and TEM images. The FTIR spectra show the presence of the polymeric chain attached to the metallic surface.

The MTT test on a normal fibroblast cell line revealed that the AgNPs stabilized with both high- and low-molecular-weight chitosan exhibited very low cytotoxicity, as compared with bare nanoparticles. The polymer-stabilized AgNPs demonstrated a significant inhibiting effect on the growth of all bacterial and fungal strains tested, considering the large dimensions of the particles.

When essential oils from thyme and clove, natural products with known antibacterial properties, were added to the colloidal dispersions, the minimum inhibitory concentration of AgNPs decreased by one or two orders of magnitude.

A systematic evaluation of the interaction of the obtained AgNPs and essential oils in various ratios was carried out in order to emphasize the appearance of synergistic effects in the antibacterial activity of the mixtures. Only an additive effect was computed between the chitosan stabilizing agent and the AgNPs. A remarkable synergistic effect was present in the chitosan-stabilized AgNP–thyme oil mixtures at the 40:1 ratio against *E. coli* and *P. aeruginosa*. A similar biological effect was evidenced in the mixtures with clove oil at 20:1 and 40:1 ratios against *E. coli* and *S. aureus*. Only an “indifferent” effect was present in the case of the antibacterial activity of chitosan-stabilized AgNP–thyme oil mixtures against *C. albicans*, while the formulation with clove oil exhibited a synergistic effect, with the maximum value recorded at the 5:1 ratio.

These results suggest that the formulations containing chitosan-stabilized silver nanoparticles and thyme or clove oils can be used as antibacterial agents for medical applications, due to their enhanced bactericidal properties and superior biocompatibility.

## Figures and Tables

**Table 1:** The MIC of bare AgNPs and AgNPs stabilized in 1% HMW chitosan solution.

Sample	MIC (ppm)			
	Tested Microbial Organisms			
	*E. coli*	*P. aeruginosa*	*S. aureus*	*C. albicans*
AgNPs	1.56	1.56	3.13	0.78
AgNPs-CH	1.56	1.56	3.13	0.78

**Table 2:** The MICs of pure essential oils.

Sample	MIC (%)			
	Tested Microbial Organisms			
	*E. coli*	*P. aeruginosa*	*S. aureus*	*C. albicans*
Thyme oil	0.25	0.25	0.50	0.50
Clove oil	0.25	0.13	0.13	0.13
